# Delayed Bronchial Obstruction following Esophageal Stent Implantation: A Case Report

**DOI:** 10.3390/medicina58020231

**Published:** 2022-02-02

**Authors:** Sang Hyun Kim, Hyuk Soon Choi, Bora Keum

**Affiliations:** Department of Internal Medicine, Korea University College of Medicine, Seoul 02841, Korea; snell17@korea.ac.kr (S.H.K.); mdkorea@korea.ac.kr (H.S.C.)

**Keywords:** esophageal stent, self-expanding metal stent, bronchial obstruction, esophageal cancer, complication

## Abstract

Airway compression is a rare complication of esophageal stent placement. With the introduction of self-expanding metal stents, the incidence of bronchial obstruction by esophageal stents has decreased. Delayed external airway compression after esophageal stent implantation is rarely reported. We describe a case of left main bronchial obstruction after self-expandable esophageal stent placement. A 70-year-old patient with advanced esophageal cancer visited the emergency room (ER) with worsening cough and dyspnea. He had received palliative concurrent chemoradiotherapy after esophageal self-expanding metal stent (SEMS) insertion three months ago. One month before the ER visit, additional esophageal SEMS placement (stent-in-stent) was performed owing to the development of a tracheoesophageal fistula. After hospitalization, chest radiography revealed a patchy consolidation in the left lower lobe. A diagnosis of pneumonia was made, and the patient was treated with antibiotics. Seven days after antibiotic treatment, the patient developed a fever and severe dyspnea. Auscultation revealed the absence of breath sounds in the left hemithorax. A follow-up chest radiograph showed a white-out of the left hemithorax. Flexible bronchoscopy revealed luminal narrowing of the left main bronchus (LMB) due to external compression. Chest computed tomography further demonstrated compression of the LMB by esophageal stents. This case highlights that esophageal SEMS can present as an emergent and often life-threatening airway obstruction.

## 1. Introduction

An esophageal stent implantation is the treatment of choice to dramatically relieve symptoms in patients with esophageal cancer. Such devices are associated with varying degrees of major complications, such as migration, tracheoesophageal fistula development, and bronchial compression [1]. To minimize these complications, rigid, inflexible plastic stents have evolved into self-expanding metal stents (SEMS) that are flexible and easy to deploy. An expandable tube of metal mesh is compressed and then inserted into the esophagus, restrained to a delivery device. Because SEMS can easily expand to a large diameter, they provide quick relief of symptoms [2]. With the introduction of self-expanding metal stents and improvements in stent quality, the incidence of bronchial obstruction by esophageal stents has decreased significantly [3]. In particular, there are few reports of bronchial obstruction occurring as a late complication of esophageal stenting [4,5]. We describe an unusual case of delayed left main bronchial obstruction that occurred a month after expandable esophageal stent placement, with a relevant literature review.

## 2. Case Report

**Figure 1:**
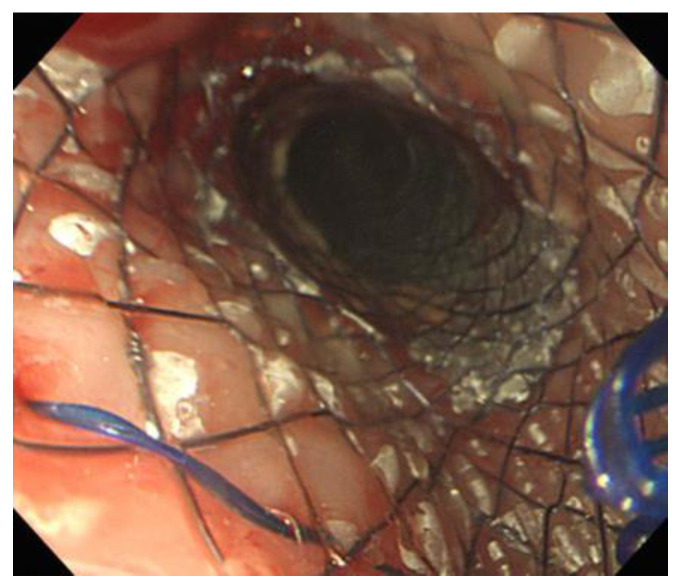
An additional esophageal self-expanding metal stent was inserted over the previously inserted stent due to the development of a tracheoesophageal fistula.

A 70-year-old man with advanced esophageal cancer (squamous cell carcinoma) presented to the emergency room for aggravated cough and breathing difficulty. The patient’s symptoms began five days before the hospital visit. His esophageal cancer was approximately 5 cm in length and located 31 cm from the incisors. Three months prior, an 8-cm long self-expanding metal stent (SEMS) was inserted into his esophagus to relieve dysphagia. The patient subsequently received palliative concurrent chemoradiotherapy. However, a month ago, additional esophageal SEMS placement (stent-in-stent) was performed (Figure 1) owing to the development of a tracheoesophageal fistula.

**Figure 2:**
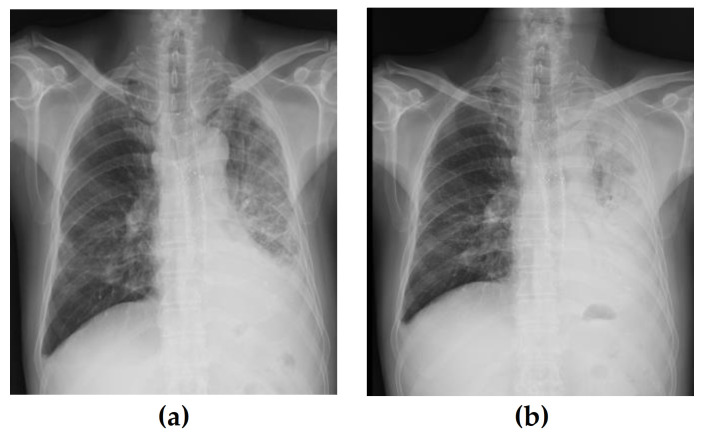
(**a**) Initial chest x-ray in the emergency room. Despite antibiotic treatment, the patient complained of worsening dyspnea. (**b**) Follow-up chest X-ray taken 7 days after hospitalization showed a white-out of the left hemithorax.

**Figure 3:**
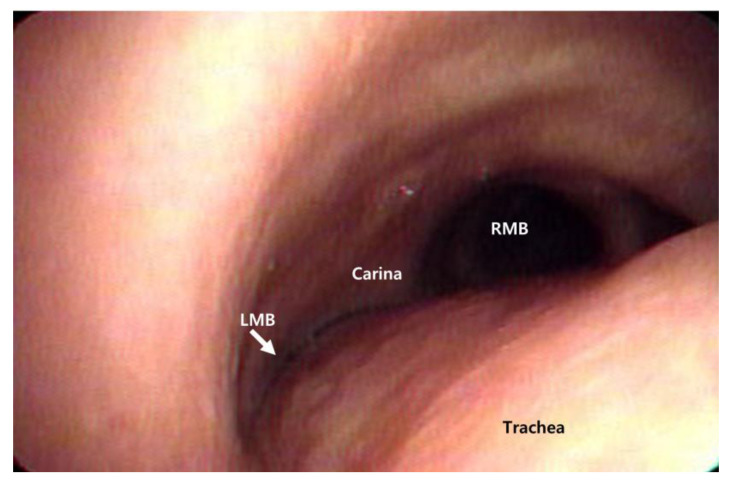
Bronchoscopic view of significant extrinsic compression of the posterior wall of trachea. The left main bronchus was also completely occluded by external compression of the esophageal stent at the site of tracheal bifurcation. LMB, left main bronchus; RMB, right main bronchus.

**Figure 4:**
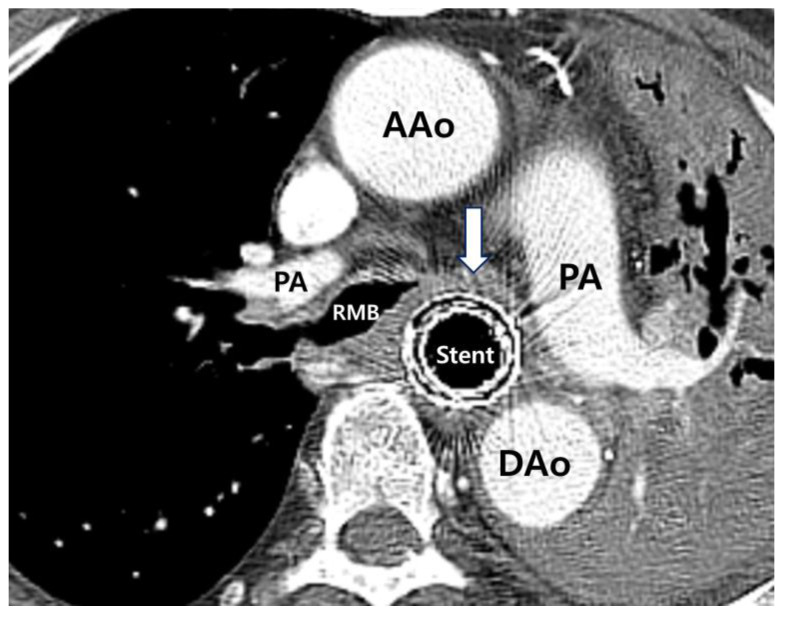
Axial view of chest CT shows severe compression of the left main bronchus (LMB) (white arrow) by the esophageal stents. Due to the self-expanding metal stents inserted into the esophagus, LMB was completely obstructed, and subsequential obstructive pneumonia occurred in the left lung. PA, pulmonary artery; RMB, right main bronchus; AAo, ascending aorta; DAo, descending aorta.

The patient’s initial temperature in the emergency room was 37.3 °C, heart rate was 93 bpm, respiratory rate was 22 breaths per minute, blood pressure was 160/100 mmHg, and oxygen saturation on room air was 94%. Blood analysis revealed mild leukocytosis (14 × 10^9^/L) with the predominance of neutrophils (86%) and normal hematocrit and platelet counts. His serum C-reactive protein level was increased to 44 mg/L (normal range, < 3 mg/L), and erythrocyte sedimentation rate was 30 mm/h. Blood biochemistry and urine analyses were normal. Chest radiography revealed a patchy increased opacity in the left lower lobe (Figure 2a). Community-acquired pneumonia was diagnosed, and the patient was treated with antibiotics. Seven days after antibiotic treatment, the patient developed a high fever and severe dyspnea. Physical examination revealed a heart rate of 110 bpm, respiratory rate of 28 breaths/min, and blood pressure of 138/88 mm Hg. Auscultation revealed the absence of breath sounds in the left hemithorax. White blood cell count was 16.07 × 10^3^/L, and serum C-reactive protein level was 183 mg/L. A follow-up chest radiograph showed a white out of the left hemithorax (Figure 2b). Flexible bronchoscopy was performed to evaluate for possible endobronchial lesions. Flexible bronchoscopy revealed marked extrinsic compression of the left main bronchus (LMB) (Figure 3). Chest computed tomography further demonstrated compression of the LMB by esophageal stents (Figure 4).

The final diagnosis in the present case was obstructive pneumonia due to delayed bronchial obstruction after esophageal stent implantation. We performed multidisciplinary expert consultations to the division of pulmonology and division of thoracic surgery to relieve the bronchial obstruction. Insertion of the bronchial stent was technically difficult to perform because the LMB was severely compressed. Surgical treatment under general anesthesia also seemed difficult, considering the patient’s systemic condition and underlying disease. After antibiotic treatment and mechanical ventilation in the intensive care unit, the patient showed some improvement. However, as the obstructive pneumonia could not be resolved, the patient died one month after hospitalization.

## 3. Discussion

Stent placement has become the treatment of choice for patients with dysphagia due to unresectable esophageal cancer [6]. Stenting of the esophagus has dramatically improved the quality of life of patients with end-stage esophageal cancer. With the development of SEMSs, complications that have been previously reported with plastic stents have decreased significantly [7,8]. Complications occurring after stent implantation can be divided into immediate and late types [3,9]. Immediate complications associated with SEMSs in the esophagus include stent misplacement, chest pain, respiratory dysfunction, and perforation [9,10]. Late complications include stent occlusion due to tumor ingrowth, food impaction, or migration [9]. The signs of respiratory dysfunction should be carefully monitored immediately after insertion. If respiratory dysfunction is observed, it is necessary to immediately exclude airway damage through bronchoscopy and assess the need for a bronchial stent [5,11]. Treatment by inserting an additional bronchial stent or removing an esophageal stent can be considered [12,13].

Delayed bronchial obstruction is a very rare complication, with few reports in the literature [4]. In our patient, after the esophageal stents were inserted, no particular signs of respiratory dysfunction were observed; however, progressive dyspnea occurred two months after implantation. The left main bronchus was severely obstructed by esophageal stents, making it difficult to insert a bronchial stent. The events that took place in our patient suggest the need for continued vigilance for the development of airway compromise.

Various risk factors for bronchial obstruction when an esophageal stent is inserted have been suggested. The most important risk factor was stent location. Because of the close anatomical relationship between the upper esophagus and tracheobronchial tree, patients with proximal esophageal tumors above the level of the tracheal carina are more likely to have airway complications from stent insertion [14,15]. A history of radiation therapy and the expansile properties of metallic stents are also important risk factors [16]. We suggest performing flexible bronchoscopy during esophageal stent placement in patients with a high risk of respiratory complications. The likelihood of airway compression by an esophageal stent can be assessed by passing a similarly sized bougie through the esophagus. If severe airway compromise is expected, it may be necessary to consider endobronchial stent placement [5,12]. If bronchoscopy is not available, it is also recommended to perform a chest CT scan after stent insertion.

The patient, in this case, had an esophageal lesion close to the tracheal carina, a history of radiation therapy, and a history of additional SEMS placement after TEF. Since the patient was at very high risk for airway complications, it would have been better to consider bronchoscopy and preventive airway stenting.

This case highlights the fact that an esophageal SEMS can present as an emergent and life-threatening delayed airway obstruction. It is important to identify adverse events immediately after stent insertion; however, the possibility of delayed adverse events should also be considered. If breathing difficulties occur at any time after esophageal stent placement, it is necessary to immediately evaluate airway compromise and assess the need for insertion of a bronchial stent using bronchoscopy.

## 4. Conclusions

In this case, sudden shortness of breath, which was thought to be unrelated to the inserted esophageal stent, was identified as a delayed complication of the esophageal stent. We believe that this case could be helpful to gastroenterologists as well as physicians in recognizing and responding appropriately to potential complications associated with esophageal stent placement.

## Data Availability

The data presented in this case report are available upon request from the corresponding author.
